# Galvanic vestibular evoked myogenic potentials: normative data and the effect of age

**DOI:** 10.1016/j.bjorl.2020.07.016

**Published:** 2020-09-17

**Authors:** Nizamettin Burak Avcı, Zahra Polat, Ahmet Ataş

**Affiliations:** a Hacettepe Üniversitesi, Faculty of Health Sciences, Audiology Department, Ankara, Turkey; b Sağlık Bilimleri Üniversitesi, Faculty of Hamidiye Health Sciences, Audiology Department, Istanbul, Turkey; c İstanbul Üniversitesi-Cerrahpaşa, Cerrahpasa Faculty of Medicine, Otorhinolaryngology Department, Istanbul, Turkey

**Keywords:** Vestibular evoked myogenic potential, Galvanic stimulation, Normalization, P1 and N1 latency, Age effect

## Abstract

**Introduction:**

Galvanic vestibular evoked myogenic potentials evaluate vestibular nerve responses using electric stimulation by records collected from the sternocleidomastoid muscle. A normal vestibular evoked myogenic potential response consists of the first positive, P1, and negative, N1, peaks. The response can be affected by factors such as age and gender and is also consequential in the diagnosis of pathologies.

**Objectives:**

The present study was performed to obtain normative data on healthy adults, to help in diagnosis by establishing clinical norms as well as to investigate changing test parameters with age in galvanic vestibular evoked myogenic potentials.

**Methods:**

A total of 100 healthy participants were included in the study. Galvanic vestibular evoked myogenic potential (current 3 mA, duration 1 ms) was performed randomly on both ears of each participant. The participants between the ages of 18–65 (mean age 39.7 ± 13.9) were divided into 5 groups according to their ages. Normative data of galvanic vestibular evoked myogenic potentials parameters were calculated in groups and in total, and age-related changes were examined.

**Results:**

The galvanic vestibular evoked myogenic potential waveform was elicited from all participants (200 ears). The latency of P1 and N1 was 7.82 ± 3.29 ms and 22.06 ± 3.95 ms, respectively. The P1−N1 amplitude value was 66.64 ± 24.5 µV. The percentage of vestibular asymmetry was 16.29 ± 11.99%. The latencies of P1 and N1 and P1−N1 amplitude values demonstrated significant differences among different age groups (*p* < 0.01).

**Conclusions:**

The results of this study show that as age increased, latencies were prolonged, and amplitudes gradually decreased. The normative data aids in the diagnosis of retrolabyrinthine lesions and the increase in the clinical use of galvanic vestibular evoked myogenic potentials.

## Introduction

Vestibular-evoked myogenic potential (VEMP) is a neurophysiological assessment method used for the evaluation of otolith organs in the inner ear. VEMPs are short-latency potentials and the inhibition response of the muscle that occurs with the stimulus.^1^ VEMP is the only vestibular test battery that evaluates the vestibulocollic reflex (VCR); therefore, it has an essential position for vestibular assessment in clinics. When considering defining applications of VEMP procedures, VEMP is used as a diagnostic tool disorders that cause dizziness, such as superior canal dehiscence, Meniere’s disease, and vestibular schwannoma.^2^ Although an acoustic stimulus is often used, a mechanical and electrical stimulus can also be performed. Different stimulation and recording modalities can be used, and different names express them, such as ocular VEMP (oVEMP), cervical VEMP (cVEMP), acoustic VEMP, etc. Galvanic vestibular stimulation (GVS) is used in the evaluation with electrical stimulus and is called galvanic VEMP (gVEMP).

The effect of the acoustic stimulus begins in the saccular macula and ends in the sternocleidomastoid muscle (SCM) via the inferior vestibular nerve, lateral vestibular nucleus, and medial vestibulospinal tract in cVEMP.^3^ GVS stimulates irregular vestibular afferents.^4^ Since GVS is an electrical stimulus, it contains anadol and cathodal currents. Cathodal current increases the spontaneous firing rate, while anodal current decreases it.^5^ This change in the spontaneous firing rate results in the stimulation of VCR. When GVS and acoustic stimuli activate the same reflex pathway from different regions, they aid in differentiating retrolabyrinth and labyrinth lesions.

VEMP results do not only show changes in diseases. Age-related vestibular degenerations also affect VCR and VEMP results. Although it has shown in many studies that vestibular degeneration is associated with age, it is difficult to determine using the results of vestibular tests.

In this study, we aimed to establish the gVEMP normative data for our country and clinic and to investigate the age-related effects of the responses in different age groups.

## Materials and methods

### Design of the study

The study was conducted in in the Hearing and Speech Disorders Center of Cerrahpasa Medical Faculty at Istanbul University-Cerrahpasa. The study was completed between November 2018 and February 2019 after approval from the ethical committee of the institute with the number 83045809-604.01.02. This study was designed and conducted in accordance with the ethical standards of the Helsinki Declaration.

The inclusion criteria for the study were: 1) Normal otoscopic and immitancemetric findings 2) pure tone average (500–2000 Hz) in the range of 0–25 dB HL 3) 18–65 years old 4) no history of vertigo and fall 5) no known neurological and orthopedic disease. A detailed explanation of the procedures that they may undergo was given to the subjects, and a signed informed consent form was obtained from each participant. Immitansmetric examinations and pure tone audiometry were performed. gVEMP was randomly applied to both ears of the participants who met the inclusion criteria.

### Participants

A hundred adults age range 18–65 (mean ± standard deviation 39.7 ± 13.9) who had no history of hearing loss, vertigo, and ear problems participated. Participants were divided into five groups according to their ages, and each group had 20 participants. The age groups were 18–25, 26–35, 36–45, 46–55, and 56–65 years, and the groups were named Group 1, Group 2, Group 3, Group 4, and Group 5, respectively. The distribution of women and men in each group was equal. A total of 50 females and 50 males participated in the study. All participants were evaluated with immitancemetric and audiological evaluations. gVEMP test was applied randomly to both ears (200 ears) of the participants.

### Immitancemetric and audiologic evaluations

The “GSI Tympstar MiddlEar Analyzer (Grason Stadler Inc., USA)” was used for the immittancemetric measurement. Tympanometry tests evaluating middle ear pressure was applied, and type A tympanogram was accepted as normal middle ear status. All hearing evaluations were performed with an “Interacoustics AC40 clinical audiometer (Interacoustics A/S, DK)”. Hearing thresholds of 500, 1000, 2000, and 4000 Hz were tested with pure tone audiometry, and pure tone averages (500, 1000, and 2000 Hz) were calculated. Hearing levels of the participants whose pure tone average was in the range of 0–25 dB HL were accepted as normal.

### Galvanic VEMP

GVS stimulated over the mastoid process causes discharge directly in the primary afferents of the vestibular nuclei and the distal part of the vestibular nerve. While the afferents on the negative (cathodal) side are activated, the afferents on the positive (anodal) side are inhibited in the bipolar configuration. In this study, the stimulating electrodes were placed according to the unilateral bipolar placement. The cathodal electrode was placed on the mastoid of the ear to be tested, and the anodal electrode was placed on the forehead. Gold cup electrodes were used for GVS. In order to prevent skin burns caused by the stimulating electrodes and to provide better conductivity, Signa electrode gel (USA) was used. The stimulating electrodes were placed on the appropriate area with the transdermal patch. When the galvanic stimulus was sent to the participants, it was stated that they would not feel pain, rather they would only feel a slight tapping.

GVS can only produce an electromyography (EMG) recording in the muscles that provide balance: EMG records from the SCM, paraspinal, triceps, tibialis anterior and soleus muscles are available. In this study, since the recording was collected from SCM, the recording electrodes were placed on the same electrode placement as the acoustic cVEMP. The active electrode, the reference electrode, and the ground electrode were placed on the middle of SCM, the sternum, and the forehead, respectively. Disposable wet-gel electrodes were used for recording. After the recording electrodes were placed, it was ensured that the impedance values were in the range of 0–5 kΩ. The ground electrode was positioned under the anodal electrode, one of the stimulus electrodes placed on the forehead with care taken to not be in contact with each other. Recording electrodes and stimulus electrodes were placed ipsilaterally on the side to be tested.

The gVEMP test was performed using Neuro-Audio (Neurosoft Inc., Russia). Neuro MEP ES control unit (Neurosoft Inc., Russia) electrical stimulator was used for GVS. The stimulus rate was 5 Hz, the stimulus duration was 1 ms, the current level was 3 mA, the stimulus waveform was a click, and the stimulus polarity was minus. For each trace, the number of stimuli was 100. Recording parameters were identical to those of acoustic cVEMP. EMG recordings were amplified for analysis. A 20–2000 Hz bandpass filter, and notch filter were applied on collected recordings. The analysis time window was 50 ms.

For the accuracy of VEMP responses, the degree of SCM muscle contraction should be stable within a specific range during the testing. In this study, this range was accepted as 30–70 μV, and the muscular tonus of SCM was measured by electromyography during testing. The feedback method of the software included in the device was used to enable the participant to maintain his/her SCM contraction.

The gVEMP test was performed with the participant in a sitting position in two stages. In the first stage, when the SCM was not contracted (head in an upright position), the first trace (without SCM contraction) was obtained by sending the galvanic stimulus over the mastoid of the side being tested. In the second stage, when the SCM was contracted (the head in rotation to the opposite direction of the ear to be tested), the second trace (with SCM contraction) was obtained by sending the galvanic stimulus. There were artefacts from the galvanic stimulus in both traces. Since these waveforms included very high artefacts, the subtraction method was used to eliminate artefacts.^6^ The first trace (without contraction of SCM) was subtracted from the second trace (with contraction of SCM), and finally, the gVEMP waveform was obtained. The same stages were conducted for both the right and left sides.

The gVEMP waveform looks the same as the acoustic VEMP waveform. The waveform has a positive and a negative peak, which are named P1 and N1, respectively. gVEMP parameters include P1 and N1 latencies, P1−N1 amplitudes, and the percentage of VEMP Asymmetry (VA). P1 and N1 latencies and P1−N1 amplitude values were recorded for each participant's right and left ear. The percentage of VA was calculated using the formula │Ar-Al│/(Ar + Al) × 100. Ar is the P1–N1 amplitude value of the right ear, Al is the P1–N1 amplitude value of the left ear, and │Ar-Al│ is the absolute value of the difference between right and left ear amplitude values.

### Statistics

The mean and standard deviation for latencies and amplitude of gVEMP and the percentage of VA were calculated. Normality tests were used to check whether or not data are normally distributed. If the skewness-kurtosis value is in the range of ±2, the data are accepted to be normally distributed.^7^ Therefore, our data was accepted to be normally distributed and parametric tests were used for all statistical analysis. Independent samples *t-*test was used for the comparison of right/left ear and female/male. One-way analysis of variance (ANOVA) test was used for the comparison between five different age groups. Post- hoc analysis was conducted to determine which group or groups had significant differences. The relationship between age and gVEMP parameters was calculated by Pearson correlation analysis. The data were analysed with IBM SPSS Statistics 20.0.

## Results

This study was carried out with 100 participants, 50 females and 50 males, and a total of 200 ears were tested. Galvanic VEMP response was obtained in all participants. P1 and N1 latencies and P1−N1 amplitude values were measured, and the percentage of VA was calculated in the study. These values were compared according to gender, ear side, and age groups.

No statistical significance was observed in the comparison of the right-left ear and male-female (*p* > 0.05). Therefore, we calculated the mean values for total and age groups. Mean values of P1 and N1 latencies, P1−N1 amplitude, and percentages of VA are shown in Table 1.

**Table 1:** Mean ± standard deviations of normative data in different age groups and total.

	P1 latency (ms)	N1 latency (ms)	P1–N1 Amplitude (µV)	Percentage of VA (%)
Group 1	6.63 ± 2.88	20.57 ± 3.16	73.39 ± 23.72	16.85 ± 14.03
Group 2	5.27 ± 1.94	20.03 ± 3.46	77.04 ± 23.55	17.91 ± 13.37
Group 3	9.04 ± 2.79	22.61 ± 3.05	68.01 ± 25.85	15.1 ± 9.32
Group 4	8.49 ± 3.46	22.61 ± 3.65	63.97 ± 19.44	11.67 ± 9.55
Group 5	9.67 ± 3.15	24.5 ± 4.68	50.77 ± 21.76	19.9 ± 12.41
Total	7.82 ± 3.29	22.06 ± 3.95	66.64 ± 24.5	16.29 ± 11.99

**Figure 1:**
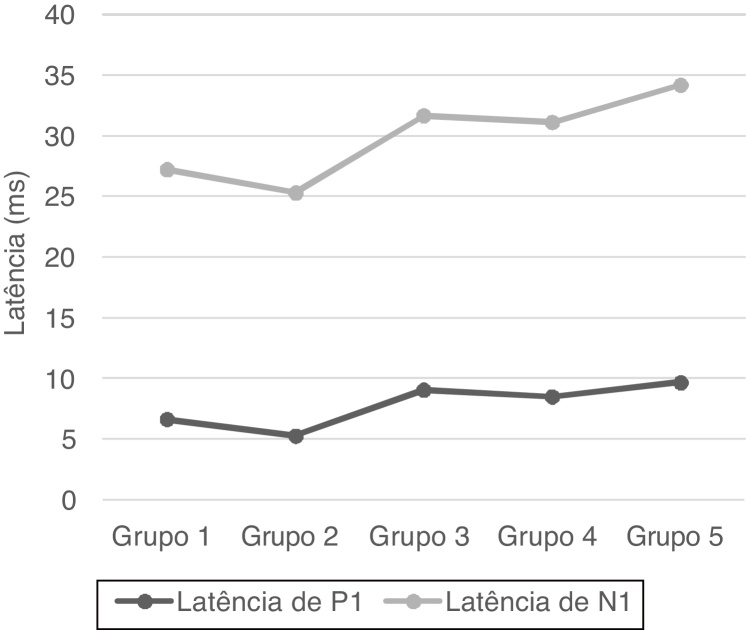
P1 and N1 latency mean values in groups.

**Figure 2:**
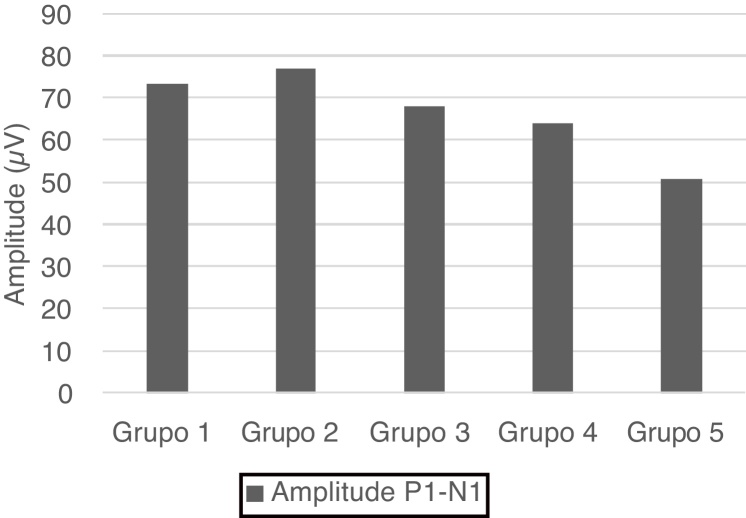
P1−N1 amplitude values in different age groups.

P1 and N1 latency values and P1−N1 amplitude values were statistically significant in the comparison between age groups. Group 1 [mean: 6.6 ms (P1) and 20.5 ms (N1)] had shorter P1 and N1 latencies than Group 3 [mean: 9 ms (P1) and 22.6 ms (N1)] and Group 5 [mean: 9.6 ms (P1) and 24.5 ms (N1)], also Group 2 [mean: 5.2 ms (P1) and 20 ms (N1)] had shorter P1 and N1 latencies than Group 3 [mean: 9 ms (P1) and 22.6 ms (N1)], Group 4 [mean: 8.4 ms (P1) and 22.6 ms (N1)] and Group 5 [mean: 9.6 ms (P1) and 24.5 ms (N1); (*p* < 0.05)] (Fig. 1). Group 5 (mean: 50.7 µV) had lower amplitude value than Group 1, Group 2 and Group 3 (means: 73.3, 77 and 68 µV respectively; *p* < 0.001) (Fig. 2). Statistical results of the comparison between groups are shown in Table 2.

**Table 2:** Comparison of between-groups for P1 and N1 latencies and P1–N1 amplitude.

Comparison of between-groups	*p*-value for P1 latencies	*p*-value for N1 latencies	*p*-value for P1−N1 amplitudes
Group 1−Group 2	0.109	0.948	0.954
Group 1−Group 3	0.003^b^	0.035^a^	0.833
Group 1−Group 4	0.081	0.070	0.358
Group 1−Group 5	0.000^b^	0.000^b^	0.000^b^
Group 2−Group 3	0.000^b^	0.006^b^	0.401
Group 2−Group 4	0.000^b^	0.015^a^	0.085
Group 2−Group 5	0.000^b^	0.000^b^	0.000^b^
Group 3−Group 4	0.933	1.000	0.934
Group 3−Group 5	0.876	0.214	0.008^b^
Group 4−Group 5	0.501	0.265	0.080

One-way ANOVA for comparison of between-groups.

Post Hocs/Tukey HSD for comparison of amplitudes.

Post Hocs/Games-Howell for comparison of P1 and N1 latencies.

a *p* < 0.05.

b *p* < 0.01.

The correlation between age and gVEMP parameters was examined. There was a weak positive correlation between age and P1 and N1 latencies (*p* <  0.001, r = 0.416 for P1 latency and r = 0.378 for N1 latency) and a weak negative correlation with P1-N1 amplitude values (*p* <  0.001, r = −0.319). No correlation was found between the percentage of VA and age (*p* =  0.873, r = −0,016).

## Discussion

Clinical research with VEMPs mostly focuses on the diagnosis of peripheral vestibular diseases. The widespread use of VEMP increases interest in the distinction between central and peripheral vestibular diseases. Therefore, different VEMP modalities should also be used to evaluate different regions of the vestibular apparatus. For instance, cervical VEMP (cVEMP) evaluates VCR and saccular function by recording on SCM, ocular VEMP (oVEMP) evaluates VOR and utricular function by recording on ocular muscles. Although abnormal cVEMP and oVEMP responses suggest a problem in the system, these tests fail to differentiate between retrolabyrinthine and labyrinthine lesions.^8^ Galvanic VEMP can be helpful in the differentiation of the lesion when used in combination with cVEMP or oVEMP because it provides stimulation bypassing the mechanical part of the inner ear.^9^, ^10^, ^11^ Therefore, the normative data obtained in our study can be used frequently in the diagnosis of the differentiation of retrolabyrinthine and labyrinthine lesions.

In our study, we applied gVEMP (3 mA, 1 ms duration) to 100 healthy individuals (200 healthy ears) between the age ages of 18–65. The P1 and N1 latencies mean values were 7.82 ± 3.29 ms and 22.06 ± 3.95 ms, respectively. The P1−N1 amplitude mean value was 66.64 ± 24.5 µV. The mean percentage of VA was 16.29 ± 11.99%. There are few studies assessing the normative data of gVEMP in the literature. In a study conducted by Cheng et al. (2018),^12^ they applied gVEMP (3 mA/1 ms duration) to 16 healthy individuals (31 healthy ears) between the ages of 20 and 60. The P1 mean latency was 11.7 ± 3 ms, the N1 mean latency was 17.8 ± 3.4 ms, the P1−N1 mean amplitude was 147 ± 69 μV, and the mean percentage of VA was 16 ± 1%. In another study conducted by Welgampola and Colebatch (2001),^13^ they obtained normative data by applying galvanic VEMP (4 mA/2 ms duration) on 70 healthy adult individuals aged 25–85 years. The mean value of P1 latency was 12.1 ± 1 ms, N1 latency was 20.2 ± 1.7 ms, P1−N1 amplitude value was 60 ± 35.1 μV, and the percentage of VA was 22%. The number of participants in these studies is less than in our study. The small differences were thought to be due to methodological differences such as recording instruments, electrode placement, and features of the stimulus and/or the differences in population.

The changes in the vestibular system occur not only in the presence of pathological conditions but also with aging. Age-related changes create effects such as a decreased number of hair cells, nerve fibers, ganglion cells, and vestibular nucleus neurons in the vestibular system.^14^, ^15^, ^16^, ^17^ In addition to the saccular macula and vestibular afferents, the accessory nerve and SCM muscle are also included in the VEMP reflex arch. The decrease in accessory nerve fibers or decreased neurogenic muscle responses should consider with aging.^18^

P1 and N1 latencies were prolonged from Group 3 and onward in our study. The prolongation of the latencies was quite significant, especially in Group 5, compared to the younger groups in this study. Although there was no statistical significance between Group 1 and Group 4 in P1 and N1 latencies, considering the mean values, we observed that group 1 had more shorter latencies than Group 4. This statistical significance was due to the difference in the standard deviations of both groups. The prolongation of P1 and N1 latencies was related to age-related changes such as the decrease in vestibular neurons, Scarpa ganglion cells, the function of myelinated nerve fibers, and the accumulation of amyloid on the vestibular nerve. We observed significantly lower amplitude values in Group 5. The lower amplitude was related to the loss of neurons due to vestibular degeneration^19^ and the degeneration of SCM.^18^ The results of this study showed that while latencies were prolonged after 35 years, the amplitude was decreased after 55 years.

The literature does not show a strong correlation between vestibular function tests and age.^20^ P1 and N1 latencies and P1−N1 amplitude values were also weakly correlated with age in our study. The correlation mirrored the results obtained in the literature. This suggests that central compensatory mechanisms have a substantial effect on the vestibular system.^21^

## Limitations of the study

Our study has a few limitations. Firstly, narrowing the age ranges in order to determine the breaking point of the age-related change can provide more specific information about vestibular degeneration. Secondly, normative data were obtained over a single current value in this study. Establishing normative data belonging to different age groups at different current values will be favorable for differentiating pathological conditions.

## Conclusion

It should be noted that differences in normalization studies may be affected by conditions such as population, stimulus type, and different test parameters. The most important contribution of this study is that it is the gVEMP normalization study with the most participants up to present and is the only study in which normative data are collected for different age groups. gVEMP, which is not yet widely used in clinics, can be used as an adjunctive diagnostic tool with acoustic VEMP in the differentiation of retrolabyrinthine and labyrinthine pathologies.

## Conflicts of interest

The authors declare no conflicts of interest.
